# Two Inexpensive and Non-destructive Techniques to Correct for Smaller-Than-Gasket Leaf Area in Gas Exchange Measurements

**DOI:** 10.3389/fpls.2018.00548

**Published:** 2018-04-24

**Authors:** Andreas M. Savvides, Vasileios Fotopoulos

**Affiliations:** Department of Agricultural Sciences, Biotechnology and Food Science, Cyprus University of Technology, Limassol, Cyprus

**Keywords:** cereals, gas exchange, LI-6400, monocotyledonous, photosynthesis, stomatal conductance

## Abstract

The development of technology, like the widely-used off-the-shelf portable photosynthesis systems, for the quantification of leaf gas exchange rates and chlorophyll fluorescence offered photosynthesis research a massive boost. Gas exchange parameters in such photosynthesis systems are calculated as gas exchange rates per unit leaf area. In small chambers (<10 cm^2^), the leaf area used by the system for these calculations is actually the internal gasket area (A_G_), provided that the leaf covers the entire A_G_. In this study, we present two inexpensive and non-destructive techniques that can be used to easily quantify the enclosed leaf area (A_L_) of plant species with leaves of surface area much smaller than the A_G_, such as that of cereal crops. The A_L_ of the cereal crop species studied has been measured using a standard image-based approach (*i*A_L_) and estimated using a leaf width-based approach (*w*A_L_). *i*A_L_ and *w*A_L_ did not show any significant differences between them in maize, barley, hard and soft wheat. Similar results were obtained when the *w*A_L_ was tested in comparison with *i*A_L_ in different positions along the leaf in all species studied. The quantification of A_L_ and the subsequent correction of leaf gas exchange parameters for A_L_ provided a precise quantification of net photosynthesis and stomatal conductance especially with decreasing A_L_. This study provides two practical, inexpensive and non-destructive solutions to researchers dealing with photosynthesis measurements on small-leaf plant species. The image-based technique can be widely used for quantifying A_L_ in many plant species despite their leaf shape. The leaf width-based technique can be securely used for quantifying A_L_ in cereal crop species such as maize, wheat and barley along the leaf. Both techniques can be used for a wide range of gasket shapes and sizes with minor technique-specific adjustments.

## Introduction

The process of photosynthesis is the key source of energy for life on earth (Zhu et al., 2008; Niinemets et al., 2017). The rapid global climate change and the arising pressure for food security (Tilman et al., 2011) are synergistically raising two important challenges to the photosynthesis research community.The first challenge is to understand the mechanisms, vulnerabilities and potentials for the improvement of the photosynthetic process and the second to develop better techniques for monitoring, modeling and rapid screening of photosynthesis (Niinemets et al., 2017).

**Figure 1:**
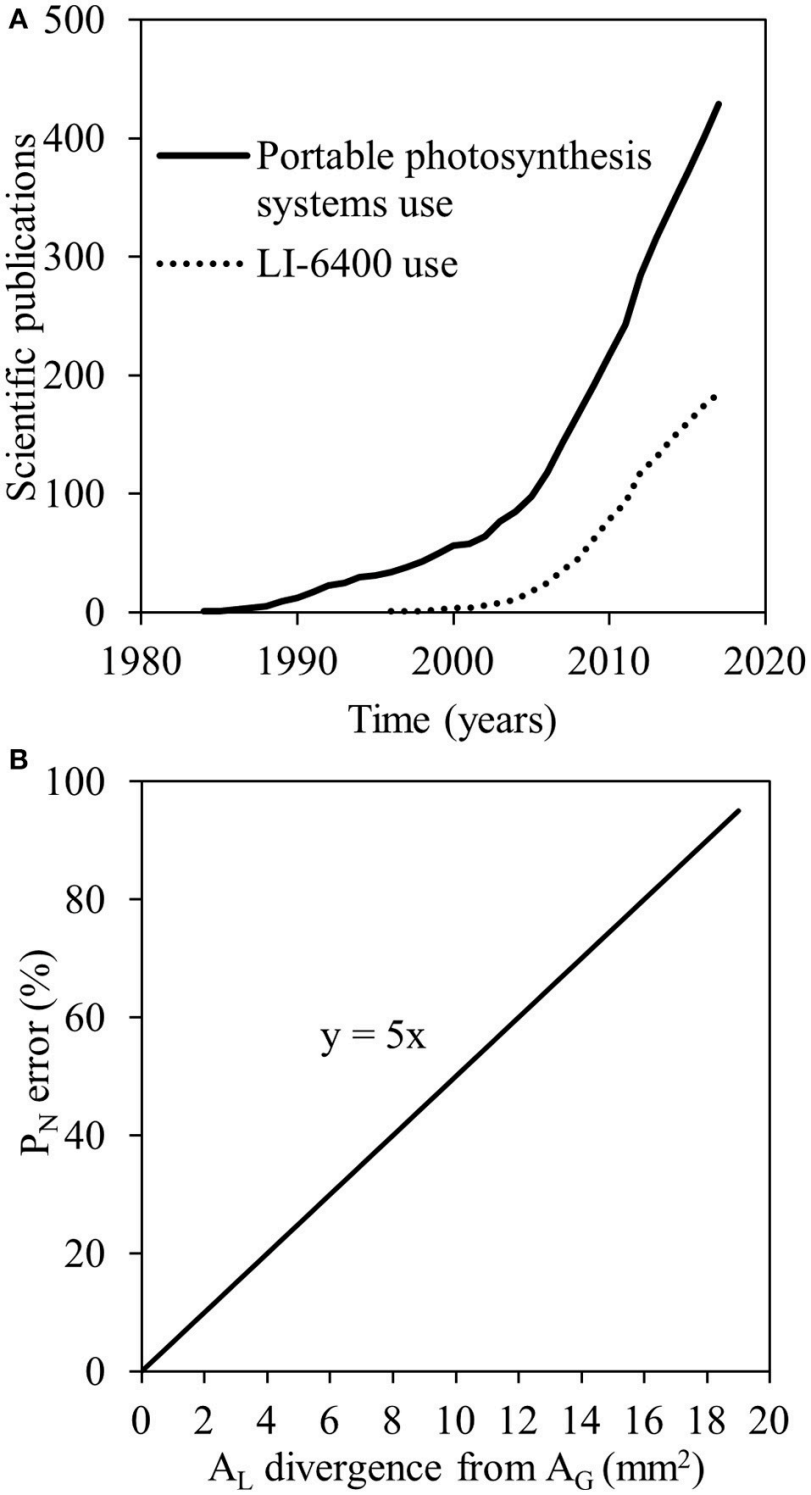
Numbers of scientific publications using portable photosynthesis systems (solid line) and LI-6400 (dashed line) based on searches performed in the Scopus database on January 08, 2018 **(A)**; the error in net photosynthesis (P_N_) calculation when the actual area of the leaf enclosed in the leaf chamber (measured leaf area; A_L_) is deviating from internal gasket area (A_G_) but A_L_ is by default considered equal to A_G_ (20 mm^2^; **B**).

The development of technology for the quantification of leaf gas exchange rates (Long et al., 1996) and chlorophyll fluorescence (Maxwell and Johnson, 2000) have offered photosynthesis research a massive boost (Long and Bernacchi, 2003). Focusing on gas exchange measurements, an increasing number of scientific publications have made use of portable photosynthesis systems over the last decades (Figure 1A). For instance, the LI-6400 portable photosynthesis system (LI-COR Biosciences, Lincoln, NE, USA) has been used in a large proportion of these studies (Figure 1A) for a wide range of applications, e.g., for the *in vivo* quantification of planar leaf photosynthetic parameters in different plant species with different leaf shapes (Savvides et al., 2012; Velez-Ramirez et al., 2014; Kaiser et al., 2016; Zait et al., 2017) grown/adapted in different environments (Velez-Ramirez et al., 2014; Kaiser et al., 2016; Rabert et al., 2017), or even for gas exchange of different plant structures (Apple et al., 2000; Savvides et al., 2013, 2014).

Over the last years, potential pitfalls of these off-the-shelf systems and ways to avoid them were displayed in literature to support a more precise quantification of photosynthetic parameters (e.g., Long and Bernacchi, 2003), for instance, the diffusional leakages of CO_2_ and H_2_O through the clamp-on leaf chambers (Flexas et al., 2007; Rodeghiero et al., 2007; Kitao et al., 2017), the air pressure changes in the leaf chamber (Jahnke and Pieruschka, 2006), the chamber response time (Weiss et al., 2009) and the overestimation of dark respiration rates because of the leaf chamber gasket shade effect (Pons and Welschen, 2002) were found to be contributing to errors when not considered in the estimation of leaf gas exchange parameters.

Gas exchange parameters (e.g., net photosynthetic rate [P_N_] and stomatal conductance [g_s_]) in such photosynthesis systems are calculated as gas exchange rates per unit leaf area (e.g., LI-COR Inc., 2015). In small chambers (<10 cm^2^), the leaf area used by the system (as a default value) for these calculations is actually the internal gasket area (A_G_) provided that the leaf covers the entire A_G_ (Long et al., 1996). Therefore, small chambers are suitable for plant species with planar leaves of surface area larger or equal to the A_G_. Completely filling the A_G_ with a leaf eliminates the error associated with leaf area determination (Long et al., 1996). There are, however, plant species with leaves of surface area smaller than the A_G_ of the smallest widely-used chambers available. For example, small-leaf monocotyledonous species (e.g., wheat) or dicotyledonous species (e.g., Arabidopsis) are hard to be measured even in such small leaf chambers. Even though narrow leaf chambers have been developed, measuring errors have been found when the latter are used because of the edge effects degrading the light field and air flow (Long et al., 1996). Due to the large errors that can derive when neglecting the correction for leaf area in small leaf gas exchange measurements (Figure 1B; Long et al., 1996), there is a great necessity for the precise quantification of the leaf area enclosed in the leaf chamber and thus measured for photosynthesis (measured leaf area; A_L_).

Different techniques were suggested in the past to measure A_L_ but they were either destructive and time-consuming (LI-COR Inc., 2015), or they are applicable only for rectangular chambers (Long et al., 1996). Gas exchange chambers are available in a variety of shapes, dimensions and functional capacity. Leaf chamber fluorometer 6400-40 (LI-COR Biosciences, Lincoln, NE) is a circular chamber that is widely-used since it is the only available one to simultaneously measure gas exchange and chlorophyll fluorescence using LI-6400. Consequently, in this report we present two inexpensive and non-destructive techniques that can be used to quantify smaller-than-gasket leaf area for leaf gas exchange measurements in circular chambers (or in chambers of other shapes after adjustments).

## Methods

### Plant material and growth conditions

Seeds of maize (*Zea mays* L. inbred line B73), barley (*Hordeum vulgare* L. cultivar “Morfo”), hard wheat (*Triticum turgidum L*. subsp. *durum* cultivar “Ourania”) and soft wheat (*Triticum aestivum* L. cultivar “Gavdos”) plants were sown in plastic pots (0.52 l volume) with potting soil (Plantaflor® potting soil, DE) and 20 germinated seedlings (one per pot) per species were grown in a climate room at 24/22°C day/night air temperature, 55–65% relative humidity and ~400 μmol mol^−1^ ambient CO_2_ concentration. During growth, plants were illuminated by fluorescent tubes at a photosynthetic photon flux density of ~100 μmol PAR m^−2^ s^−1^ during 16 h photoperiod. All the measurements were performed on the first true leaf 2 weeks after germination.

### Photosynthetic measurements

**Figure 2:**
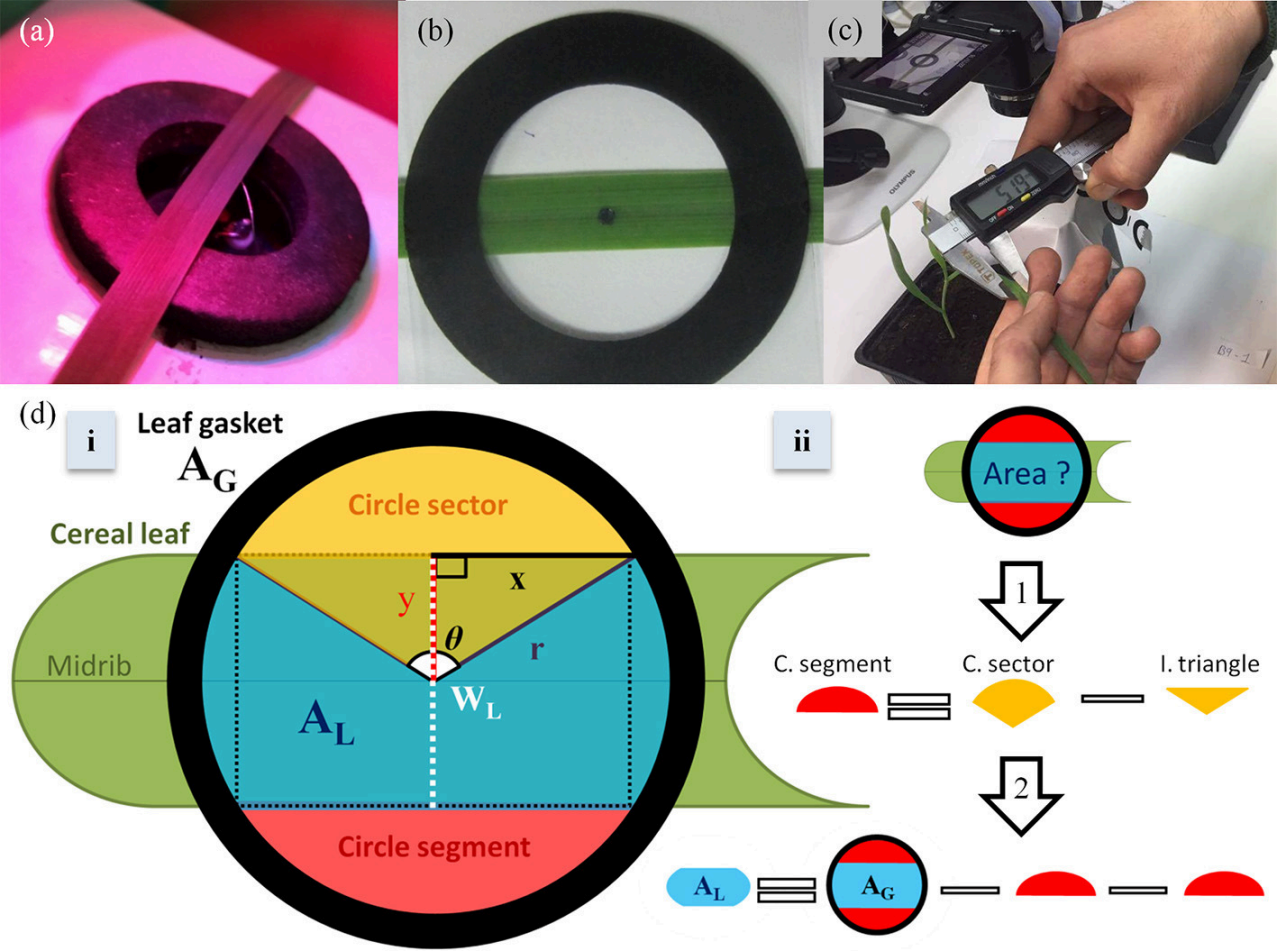
A wheat leaf smaller than the leaf gasket internal area **(a)**; The impression of the leaf chamber used to image and quantify measured leaf area (*i*A_L_; **b**); measurement of leaf width in wheat plants using an electronic caliper **(c)**; a scheme of the concept based on which the equation for calculating measured leaf area (*w*A_L_) based on leaf width (W_L_) was built **(d)**. Under the assumption that the empty space created by the enclosure of a cereal leaf with leaf width (W_L_) smaller than the internal gasket diameter (2r) in the middle of the chamber is creating two equal circle segments (ii), the *w*A_L_ will be equal to the internal gasket area (A_G_) minus the two empty circle segments (ii). Considering that A_G_ is known and that the circle segment is a part of a circle sector that is comprised of a circle segment and an isosceles triangle (i), the circle segment area can then be calculated by the subtraction of the isosceles triangle from the circle sector (ii: arrow 1). The circle sector area and the isosceles triangle area can be calculated based on known dimensions (*y* = W_L_/2, r and x) and the angle θ (i).

The measurements of leaf gas exchange, g_s_ and P_N_, and operating efficiency of photosystem II (Φ_PSII_; Baker, 2008) were performed using a portable gas exchange system LI-6400XT (LI-COR Biosciences, Lincoln, NE, USA) combined with a leaf chamber fluorometer 6400-40 (LI-COR Biosciences, Lincoln, NE). Intrinsic water use efficiency (iWUE) was then calculated as the ratio between P_N_ and g_s_. Microclimatic conditions were adjusted in the leaf chamber to be similar with those of the growth environment. Light intensity in the leaf chamber was set at 100 μmol m^−2^ s^−1^ in all cases, while spectral quality was set at 90/10% red/blue light mixture. Leaf chamber temperature was stable at 24°C and the CO_2_ reference concentration was 400 μmol mol^−1^. Relative air humidity was controlled at ~60%. All measurements were carried out inside the growth rooms. In total, ten plants were used for gas exchange measurements per plant species. The midrib of the part of the middle leaf blade measured was placed in the center of the leaf chamber (Figure 2a) to allow the precise forthcoming quantification of A_L_. The above measurements were repeated in the same environment on dead (dried) leaves of every species to identify and correct for possible effects of A_L_ on CO_2_ diffusional leakage.

### Leaf area measurements

A_L_ was quantified using (i) imaging in combination with an image processing program (image-based A_L_; *i*A_L_) and (ii) estimated based on a single leaf width (W_L_) measurement and geometry (width-based A_L_; *w*A_L_).

#### Image-based technique

Exact impressions of the chamber gasket (Figure 2a) were designed in Microsoft® Powerpoint™ and printed on transparent film (Figure 2b). Outside the impression, a black bar of known distance (1 cm) was printed to later allow the scale setting in the software used. Following leaf gas exchange measurements, the leaf was taken out of the leaf chamber and horizontally but gently placed below the impression and above a white paper sheet to allow contrast for the image processing. Caution was taken to firstly leave the plant and the measured leaf undamaged and secondly position the part of the leaf measured in LI-6400XT at the exact same place under the impression (Figure 2b). The measured part of the leaf blade including the gasket impression were imaged (Figure 2b) and the image was processed using ImageJ (open access software, version 1.50b) as described below.

Image processing steps: (1) A selected image is opened in ImageJ, (2) the “straight line” tool is selected and then a straight line is drawn along the black scale bar of the image, (3) a scale is set by selecting “analyze” and then “set scale,” (4) in the newly opened window the value 1 (cm) is typed as a “known distance,” (5) after clicking “ok,” the “freehand selections” tool is selected and a freehand line is drawn forming a shape that is carefully and tightly enclosing only the part of the leaf area included in the black circle formed by the impression of the leaf gasket (Figure 2b), (6) *i*A_L_ is quantified by selecting “analyze” and then “measure,” (7) the value (in cm^2^) then appears in a new window.

#### Leaf width-based technique

*w*A_L_ estimation was based on the A_G_, the leaf surface geometry and W_L_. It was assumed that the two margins of a monocot leaf surface are parallel straight lines with equal distance from the main leaf midrib (y; Figure 2d). Based on this assumption, a monocot leaf inserted and positioned in the center of the circular leaf chamber, that has a W_leaf_ smaller than the internal diameter of the chamber, will cover only a part of A_G_ shaping in that way two equal unfilled (by the leaf) circle segments (Figure 2d). *w*A_L_ can therefore be easily quantified by subtracting the two unfilled circle segments from A_G_. A_G_ is known (e.g., for 6400-40 Leaf Chamber Fluorometer is 2 cm^2^). The area of the circle segment can be quantified when the latter is considered a part of a circle sector (Figure 2d). The circle sector is comprised of the circle segment and an isosceles triangle created by the two equal straight lines (i.e., radius) connecting the two angles of the circle segment with the center of the circle. The area of the circle sector can be quantified using the equation:

(1) $$\begin{array}{r} Circle\;sector\;area=\pi r^{2}\frac{\theta^{{}^{\circ}}}{360^{{}^{\circ}}} \end{array}$$

Where r is the radius of the circle, θ is the central angle of the sector and π is the mathematical constant (π = 3.14159).

*r* can be calculated using the equation:

(2) $$\begin{array}{r} r=\sqrt{\frac{A_{G}}{\pi }} \end{array}$$

θ, the central angle of the circle sector can be calculated using the following reasoning: y is the straight line connecting the center of the circle with the middle of the straight line shaping the circle segment (i.e., leaf margin; Figure 2d) and therefore, y is a known (measurable) distance as it is the half of W_L_. Additionally, y is the adjacent (to θ/2 angle) side of the two identical right-angled triangles shaped by r (i.e., hypotenuse), y, and x which is the opposite (to θ/2 angle) and the half of the length of the enclosed leaf margin (Figure 2d). θ/2, the angle produced by y and r, is actually the half of the central angle of the circle sector, can then, be calculated based on y and r using the following equation:

(3) $$\begin{array}{r} \theta /2=\left[\cos^{-1}\left(\frac{y}{r}\right)\right]\left(\frac{180^{o}}{\pi }\right) \end{array}$$

Therefore, θ is calculated using the following equation:

(4) $$\begin{array}{r} \theta =2\left[\cos^{-1}\left(\frac{y}{r}\right)\right]\left(\frac{180^{o}}{\pi }\right) \end{array}$$

This isosceles triangle is comprised of two identical right angled triangles of known dimensions. Therefore, the area of the triangle can be calculated using the following equation:

(5) $$\begin{array}{r} Isosceles\;triangle\;area=y\sqrt{r^{2}-y^{2}} \end{array}$$

After calculating the circle sector area using r and θ (Equation 1), circle segment area can be calculated by subtracting the area of the isosceles triangle from the total circle sector area (Equation 6; Figure 2d).

(6) $$\begin{array}{r} Circle\;segment\;area=\;\pi r^{2}\frac{\theta^{{}^{\circ}}}{360^{{}^{\circ}}}-\;y\sqrt{r^{2}-y^{2}} \end{array}$$

*w*A_L_ can then be calculated by subtracting the two identical unfilled circle segments from A_G_ (Figure 2d). Substituting r and θ with their equation equivalents (see Equation 2 and Equation 4 respectively) and y with W_L_/2 to have only known parameters in a final *w*A_L_ equation (e.g., in this study, A_G_ equals 2 cm^2^), *w*A_L_ can be calculated when measuring only W_L_ by the following equation:

(7) $$\begin{array}{r} w{\text{A}}_{\text{L}}={\text{A}}_{\text{G}}-\frac{2{\text{A}}_{\text{G}}\left[\cos^{-1}\left(\frac{{\text{W}}_{\text{L}}}{2\sqrt{\frac{{\text{A}}_{\text{G}}}{\pi }}}\right)\right]}{\pi }+{\text{W}}_{\text{L}}\sqrt{\frac{{\text{A}}_{\text{G}}}{\pi }-\frac{{{\text{W}}_{\text{L}}}^{2}}{4}} \end{array}$$

**Figure 3:**
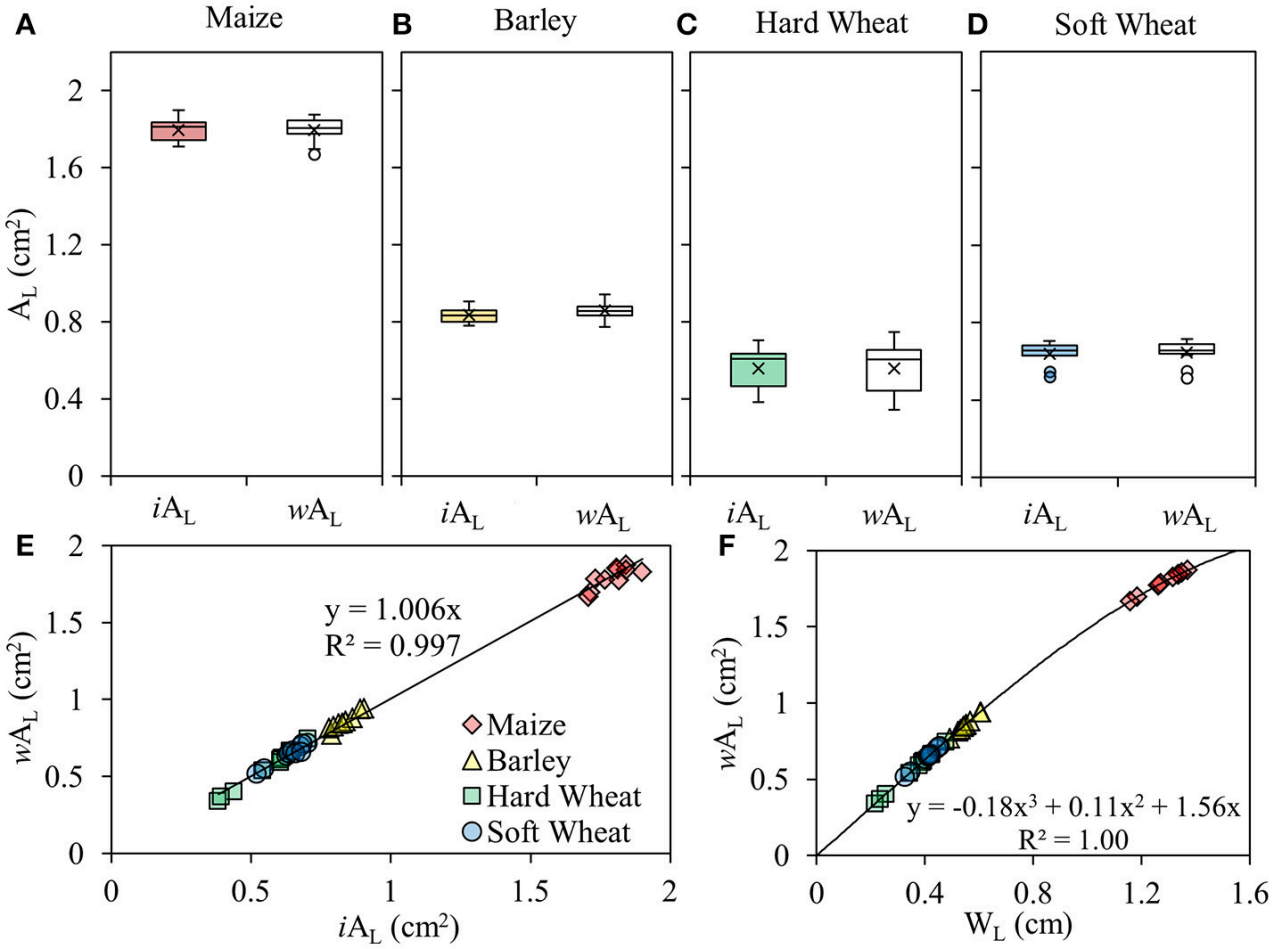
Box plots of the leaf area included in the leaf gasket during photosynthesis measurements (A_L_) measured using an image-based approach (*i*A_L_) and estimated using a leaf width–based approach (*w*A_L_) of maize **(A)**, barley **(B)**, hard wheat **(C)** and soft wheat leaves **(D)** (*P* < 0.05; *n* = 10). The lower and upper part of the boxes give the estimated 25 and 75th percentile, the horizontal line through the box indicates the median value, the x markers indicate the mean of the sample, the top of upper whisker indicates the maximum value of the sample and the bottom of the lower whisker indicates the minimum of the sample; the linear relation between the *w*A_L_ and *i*A_L_ **(E)**; the curvilinear relation between the W_L_ and *w*A_L_ **(F)**.

W_L_ was quantified at the middle of the segment of the enclosed leaf blade (Figure 2d) using an electronic caliper (31C628, TOPEX, PL; measurement accuracy 0.01mm; Figure 2c). The relation between W_L_ and *w*A_L_ is curvilinear (Figure 3F).

Despite the measurements on the middle blade that were related to the photosynthetic parameters measured in this study, A_L_ quantification using both the techniques was performed in different positions along the leaf blade to investigate whether *w*A_L_ can be successfully used not only on the middle leaf blade but along the leaf regardless the changes in leaf shape.

### Statistics

The data were analyzed using the statistical analysis software package IBM SPSS Statistics 20 (IBM Corporation, US). A paired *t*-test was used to compare the *i*A_L_ and *w*A_L_ (*P* < 0.05). The samples compared were tested for normality using Shapiro-Wilk test (*P* < 0.05). Two-way analysis of variance (ANOVA) and post hoc Tukey's honestly significant difference (HSD) multiple comparison tests (*P* < 0.05) were used to evaluate statistically significant effects (*P* < 0.05) of (1) the factors “plant species” and “position on the leaf” and their interaction on the divergence between *w*A_L_ and *i*A_L_ and (2) the factor “plant species” and the covariate “*i*A_L_” and their interaction on Φ_PSII_. One-way analysis of variance (ANOVA) and *post hoc* Tukey's honestly significant difference (HSD) multiple comparison tests (*P* < 0.05) were used to evaluate statistically significant differences on the P_N_, g_s_, Φ_PSII_ and iWUE between the species studied.

## Results

### Measured leaf area

The A_L_ of the monocotyledonous species studied has been measured using a standard image-based approach (*i*A_L_) and estimated using a leaf width-based approach (*w*A_L_; Equation 7). *i*A_L_ and *w*A_L_ did not show any significant differences between them in maize (Figure 3A), barley (Figure 3B), hard wheat (Figure 3C) and soft wheat (Figure 3D). Based on these results, a strong linear relation (*Y* = 1.006X, *R*^2^ = 0.997) was developed between *i*A_L_ and *w*A_L_ when the data points from all the species used in this study were included (Figure 3E).

**Figure 4:**
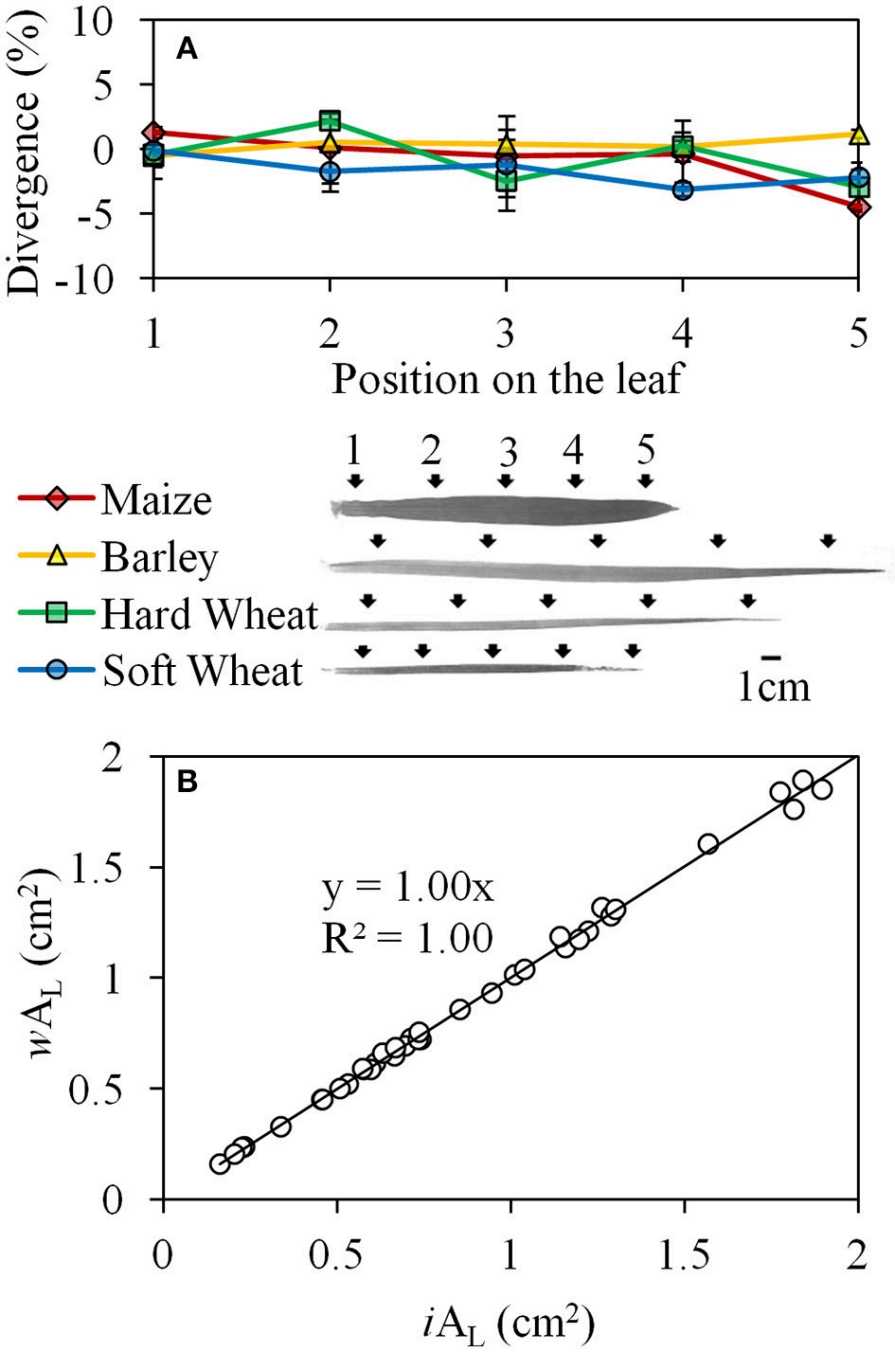
The divergence (%) of *w*A_L_ from *i*A_L_ when A_L_ was quantified on five different positions (black arrows) along the leaves of maize, barley, hard and soft wheat **(A)**; the linear relation between *i*A_L_ and *w*A_L_ when the data points from all the species and the positions on the leaf were incorporated **(B)**. Error bars indicate the standard error.

Similar results were obtained when the *w*A_L_ was tested in comparison with *i*A_L_ along the monocotyledonous leaf in all species studied (Figure 4). The divergence of *w*A_L_ from *i*A_L_ did not exceed ±5% and was not significantly influenced by the plant species (*P* = 0.265) or the position of the measurement on the leaf (*P* = 0.244), while no interaction was found between the factors “species” and “position on the leaf” (*P* = 0.480; Figure 4A). Based on these results, a strong linear relation (*Y* = X, *R*^2^ = 1) was developed between *i*A_L_ and *w*A_L_ when the data points from all the species and the positions on the leaf were incorporated (Figure 4B).

### Photosynthetic parameters

**Figure 5:**
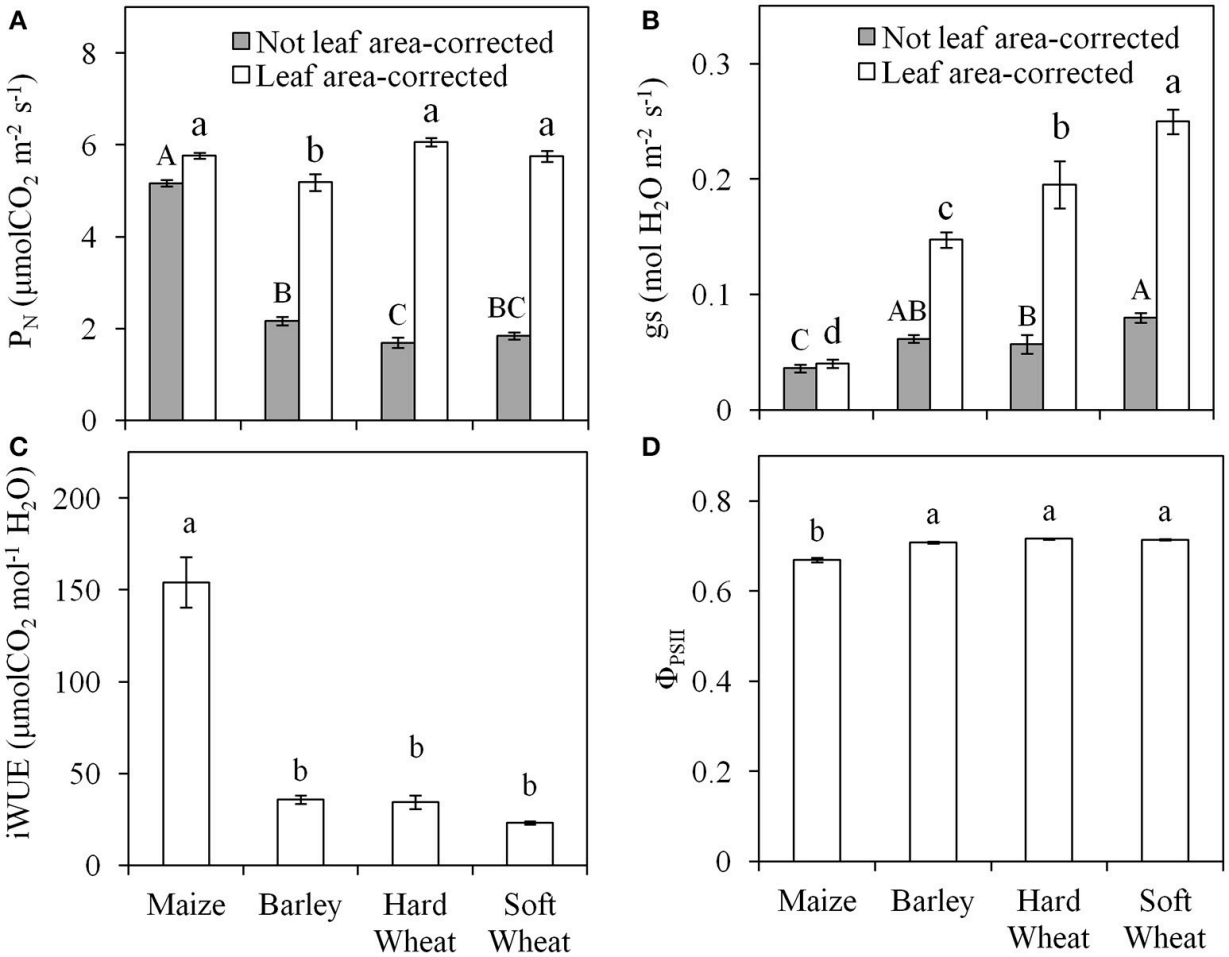
The values of net photosynthetic rate (P_N_; **A**) and stomatal conductance (g_s_; **B**) when not-corrected for the leaf area measured (gray bars) and when corrected for leaf area measured (white bars); intrinsic water use efficiency (iWUE= P_N_/g_s_; **C**) and operating efficiency of photosystem II (Φ_PSII_; **D**) of the first true leaf of maize, barley, hard, and soft wheat. Different letters indicate significant differences (*P* < 0.05; *n* = 10). Error bars indicate the standard error.

The photosynthetic parameters have been corrected for A_L_ using the *i*A_L_ and not both *i*A_L_ and *w*A_L_ due to the similar results obtained when using either the image- or the leaf width-based technique. As expected, the level of misestimation of leaf gas exchange parameters when *i*A_L_ was not considered in their calculation was larger (Figures 5A,B) the smaller the *i*A_L_ (Figures 3A–D). The difference observed between the *i*A_L_-corrected and not *i*A_L_-corrected values of P_N_ (Figure 5A) and gs (Figure 5B) was larger for the small-leaf species (i.e., soft wheat>hard wheat>barley) and the smallest for maize. The actual (i.e., *i*A_L_-corrected) P_N_ was significantly lower in barley when compared with other species (Figure 5A), while the actual g_s_ was significantly the highest in soft wheat, lower in hard wheat and barley and the lowest in maize (Figure 5B). The iWUE was significantly higher in maize in comparison with all other plant species examined (Figure 5C). The Φ_PSII_ was significantly lower in maize (0.67) in comparison with all other plant species examined (0.71; Figure 5D). No significant effects of *i*A_L_ were observed on Φ_PSII_ (*P* = 0.083).

The level of the CO_2_ diffusional leaks observed when CO_2_ exchange was quantified without including a leaf in the leaf chamber (i.e., empty chamber) was not significantly different from the levels measured when dried leaves from each of the plant species studied were inserted in the leaf chamber (*P* = 0.22; Figure S1a). Additionally, CO_2_ diffusional leaks were not significantly correlated with A_L_ across the species (*P* = 0.12; Figure S1b).

## Discussion

### Image-based technique

Imaging techniques have a key role in plant phenotyping (Li et al., 2014). In this study, a simple and standard (in terms of image processing) imaged-based technique has been used to assist accurate leaf gas exchange measurements of narrow leaves in important cereal crop species using the portable photosynthesis system LI-6400XT. Even though the leaf chamber fluorometer 6400-40 was used here, the technique can be adjusted for measurements using different leaf chambers and photosynthesis systems by just adjusting the gasket impression respectively. The use of a gasket impression enabled a non-destructive approach that can securely replace destructive methods (e.g., LI-COR Inc., 2015). The use of a freeware, like ImageJ, for image processing and area quantification reflects a low-budget technique. Last but not least, this technique can be used to quantify A_L_ on leaves of various shapes and sizes. Consequently, the image-based technique allows the quantification of leaf gas exchange parameters on every edge of a leaf.

### Leaf width-based technique

The shape of the leaves of cereal plants is simple and thoroughly described in literature (Arber, 1923). Grass leaves consist of a sheath, a ligule and a blade. Blades, representing the main photosynthetically active leaf part, are long and narrow and they are usually linear to lanceolate (Sylvester et al., 2001). The leaf shape, even though not largely influenced by the growth conditions, is highly dependent on the genotype and ontogeny (Sylvester et al., 2001; Dornbusch et al., 2011).

The leaf width-based technique developed in this study is applicable along the leaf and for all the genotypes tested despite the longitudinal and species-related differences in leaf shape (Dornbusch et al., 2011). Supported by these results, the single W_L_ measurement through the center of the circular leaf gasket was adequate for the precise *w*A_L_ quantification based on the equation developed (Equation 7).

A similar approach was suggested in the past (Long et al., 1996), but it is possibly effective only when measuring with a rectangular leaf chamber. According to Long et al. (1996), if the leaf shape allows the user to assume that the enclosed tissue forms a trapezium, then the area enclosed may be determined simply by measuring the leaf widths (i.e., trapezium bases) at each end (Long et al., 1996). However, in the case of a circular gasket, the enclosed tissue does not form a simple trapezium but a compound shape that it is more like a combination of a rectangle or trapezium with its two bases attached to circle segments. Therefore, adopting the leaf width-based technique instead of the “single trapezium” theory for circular gaskets would result in avoiding an underestimation of A_L_.

For the equation development, it was assumed that the two margins of a monocot leaf surface are parallel straight lines with equal distance from the main leaf midrib. In reality, the two margins of the leaf blades of the cereals tested are not straight lines and definitely not parallel (Dornbusch et al., 2011). However, the equation developed based on W_L_ can be successfully applied in both the cases (i.e., parallel or not) and the reason is simply that W_L_ represents the median (i.e., the parallel line segment half-way between the two bases) for both a rectangle and a trapezium. Considering the latter statement, the area of a rectangle and that of a trapezium depends only on their median and height (i.e., the distance between the two bases). The area of a rectangle and that of a trapezium with equal median and height will be equivalent. Consequently, at a constant W_L_, width changes at the two edges of the enclosed leaf tissue will not be influential for A_L_. This seems to be the reasoning behind the successful application of the leaf width-based technique along the leaf blade and across grass species.

### Correction for leaf area in photosynthetic parameters

The quantification of A_L_ and the subsequent correction of leaf gas exchange parameters for A_L_ provided a precise quantification of P_N_ and g_s_ especially with decreasing A_L_. Under the conditions studied, maize showed the highest P_N_, the lowest g_s_ and therefore the highest iWUE in agreement with the statement that C_4_ plants, like maize, have a higher iWUE than C_3_ plants, like wheat and barley (Taylor et al., 2010). The higher iWUE may be either due to lower g_s_ only (Taylor et al., 2010) or due to both higher P_N_ and lower g_s_ in C_4_ species compared to C_3_ species (Kocacinar, 2015) something that was also observed in this study. Φ_PSII_ was significantly lower in maize than in the other species studied. Φ_PSII_ at a given photosynthetic photon flux density provides an estimate of the quantum yield of linear electron flux through photosystem II (Baker, 2008) and can be different between C_3_ and C_4_ species depending on the growth conditions (Oberhuber et al., 1993; Wang et al., 2015). Additionally, no effects of A_L_ on Φ_PSII_ were detected suggesting that chlorophyll fluorescence parameters are not influenced when the leaf gasket is not completely covered by the leaf.

Changes in A_L_ and the plant species did not have any significant effects on the diffusional CO_2_ leakages through the leaf chamber under the conditions used in this study. This may be due to the fact that the CO_2_ gradient between the chamber and the surrounding air was rather small because the measurements took place under controlled - similar to the growth room - conditions. It is known that major diffusional leakages are significant at large CO_2_ gradients and that they are species-dependent (Flexas et al., 2007).

## Conclusions and future perspectives

This study provides a practical, inexpensive and non-destructive solution to researchers dealing with photosynthesis measurements on small-leaf plant species. The image-based technique can be widely used for quantifying A_L_ in many plant species despite their leaf shape. Additionally, the technique can be easily adjusted for measurements using leaf chambers of other shapes (e.g., rectangular) by just adjusting the gasket impression respectively. Image analysis for leaf area determination is being widely and frequently used in studies (e.g., Bylesjö et al., 2008; Maloof et al., 2013). We have here used a very easy but time-consuming way to extract A_L_ from image analysis using ImageJ. Future studies may accelerate A_L_ determination by adopting image analysis tools (or their principles), especially developed for leaf size determination (e.g., Bylesjö et al., 2008; Maloof et al., 2013). The leaf width-based technique can be securely used for A_L_ quantification in cereal crop species such as maize, wheat and barley along the leaf, when the latter is enclosed in circular chambers. The latter can be considered as a faster and easier-to-apply (it is based on a single leaf width measurement) than the image-based technique. However, its application is restricted to leaves with shape similar to that of the cereals tested. Regarding potential diffusional leaks, future studies aiming to quantify, for example, the response of net photosynthesis to leaf internal CO_2_ (i.e., A-C_i_ curves) or gas exchange responses to vapor pressure deficit should consider quantifying possible effects of A_L_ and leaf structure on CO_2_ and H_2_O leakages from and to the leaf chamber (e.g., Flexas et al., 2007; Kitao et al., 2017).

## Author contributions

AS designed and performed the experiments, did the statistics and wrote the manuscript. VF read and commented on the manuscript. AS and VF revised the manuscript.

### Conflict of interest statement

The authors declare that the research was conducted in the absence of any commercial or financial relationships that could be construed as a potential conflict of interest.

## Abbreviations

A_G_: internal gasket area
A_L_: measured leaf area
g_s_: stomatal conductance
*i*A_L_: image-based measured leaf area
P_N_: net photosynthetic rate
*w*A_L_: leaf width-based measured leaf area
W_L_: leaf width
Φ_PSII_: operating efficiency of photosystem II.
