# Supplementary material for: Two Inexpensive and Non-destructive Techniques to Correct for Smaller-Than-Gasket Leaf Area in Gas Exchange Measurements
Source: Front Plant Sci. 2018 Apr 24;9:548. doi: 10.3389/fpls.2018.00548 (PMC5928467; doi:10.3389/fpls.2018.00548)
Supplement: Supplementary file 1 [file Data_Sheet_1.docx]

## Supplementary Material

The Supplementary Material for this article can be found online at:

## ****Figure S1****. The apparent photosynthetic rate (Apparent P_N_; not normalized for leaf area) as quantified by LI-6400XT with empty chamber or on dried leaves of maize, barley, hard and soft wheat for the quantification of potential CO_2_ diffusional leakages (a); The apparent P_N_ plotted against imaged-based measured leaf area (*i*A_L_
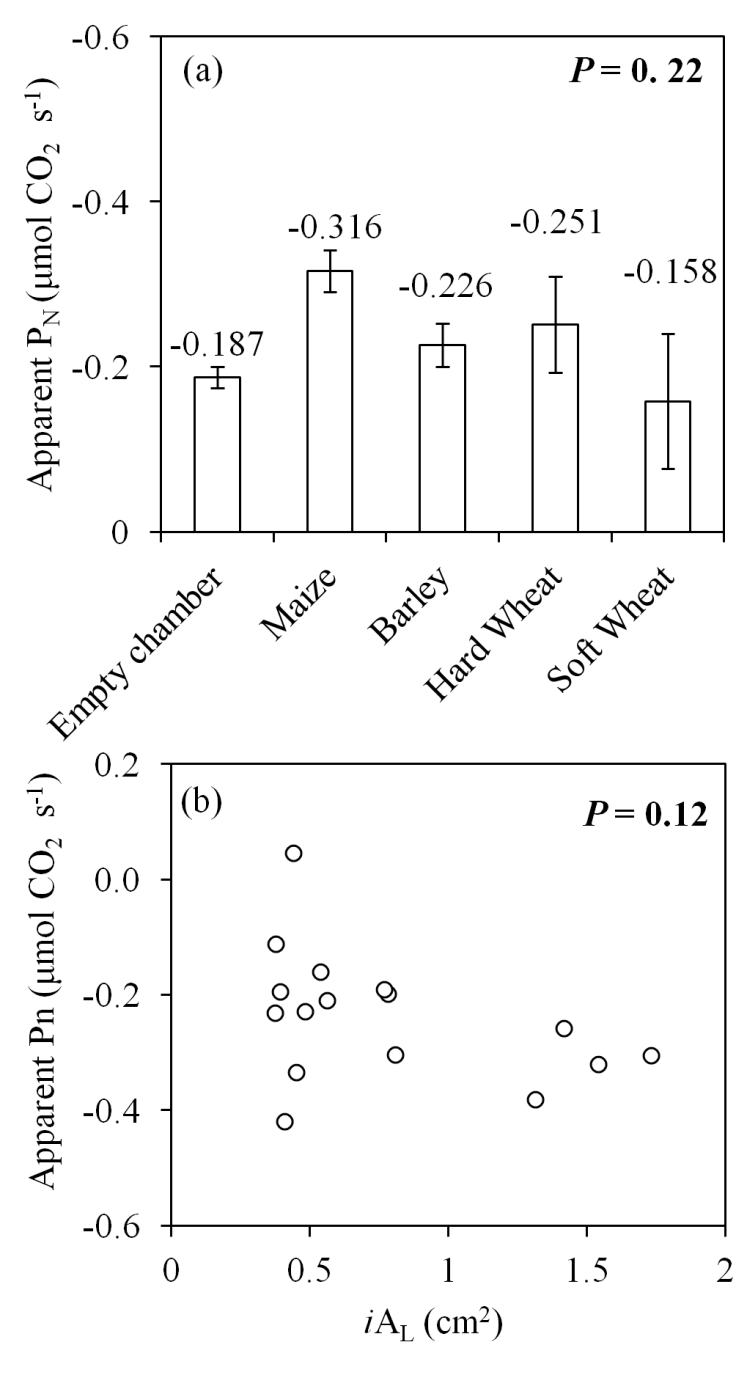
; P<0.05; n=4; b).
